# A seven-year retrospective analysis of patch test data in a cohort of patients with contact dermatitis in Sri Lanka

**DOI:** 10.1186/s12895-019-0090-8

**Published:** 2019-07-10

**Authors:** B. S. D. P. Keragala, H. M. M. T. B. Herath, T. S. Keragala, M. A. M. H. Malavi, Chaturaka Rodrigo, C. N. Gunasekera

**Affiliations:** 1 National Hospital of Sri Lanka, Colombo, Sri Lanka; 2 Department of Pathology, School of Medical Sciences, University of New South Wales, Sydney, 2052 Australia

**Keywords:** Patch test data, Contact dermatitis, Sri Lanka

## Abstract

**Background:**

Patch testing with a baseline series is a common tool employed when the sensitizing agent in contact dermatitis is unclear. However, for Asian countries, there are no locally validated baseline series to utilize in screening.

**Methods:**

We completed a retrospective analysis of all patients that had undergone patch testing with the European Baseline series, Shoe Series or Comprehensive International Baseline series, over 7 years from 2012 to 2018 in a tertiary care reference dermatology clinic in Sri Lanka to evaluate the suitability of these investigations to identify causes for contact dermatitis in the local study population.

**Results:**

Out of 438 patients tested, 239 (54.8%) reacted to at least one substance in the series. The Shoe Series was significantly more likely to yield a positive result than the European Baseline Series (70.2% vs 46.9%, *p* < 0.05). The top three sensitizers identified by all series were nickel sulfate (16%, 70/438), p-phenylenediamine (12.3%, 54/438) and 2-mercaptobenzothiazole or mercapto mix (10.5%, 46/438).

**Conclusion:**

Shoe series has a comparatively high yield in the local population compared to European Baseline series. Since little less than half of the study population did not have any reactivity to any of the allergens tested it is important to develop or modify and validate a locally relevant, more suitable baseline series which is based on the Shoe Series in Sri Lanka. This is further evidence for the continuously changing nature of allergens in the environment and the need to modify existing patch testing standards accordingly.

**Electronic supplementary material:**

The online version of this article (10.1186/s12895-019-0090-8) contains supplementary material, which is available to authorized users.

## Background

Contact dermatitis is characterized by an acute or chronic inflammatory response to an irritant (irritant contact dermatitis) or an allergenic substance (type IV delayed type hypersensitivity). Contact allergies are usually caused by chemicals with a molecular weight of < 500 Da, but exceptionally also be between 500–1000 Da. These chemicals, when bound to proteins serve as antigens that sensitize a susceptible individual who will subsequently mount a secondary immune response during repeated exposures [1]. In addition to the primary sensitizing allergen, cross-sensitivity can also occur with structurally related allergens without prior sensitization. In United States, the total medical cost due to contact dermatitis was estimated to be around $1529 million in 2017. The total productivity lost is estimated to be around $700 million [2]. Despite the medical costs being different in other parts of the world, given the prevalence of the condition, it may still account for a significant loss of productivity in any country, especially when affected individuals have to change their jobs. In Sri Lanka, contact dermatitis is frequently diagnosed in family practices, adult health or dermatology clinics but its disease burden remains under-appreciated.

Patch testing is the standard procedure used to diagnose contact allergy in a patient with a history of dermatitis. Pre-packaged tests are available, with a limited number of allergens determined by research and experience of experts in working groups. Such series as the European Baseline Series (EBS), Shoe Series (SS) and International Comprehensive Baseline Series (ICBS) have various allergens some of which get updated based on continuous surveillance. However, it is not sufficient to patch test with only the suspected allergen, as it may not be the cause. Sometimes a number of allergens, mainly fragrances and rubber compounds, are compiled into mixes to save space. When a positive reaction occurs to a mix, such as a fragrance mix, a subsequent breakdown test using its constituent ingredients is carried out. If all of this is inadequate, additional patch tests are carried with customized lists or series, according to the history of exposures of the patient. Thus any baseline series used routinely for patch testing needs to be revisited over time as the allergens keep on changing in a population. Likewise, the exposure to triggering agents may also differ between different geographic regions and therefore one series designed with data from a particular region may not be the best fit for another. Yet, in the absence of alternative data, in developing countries like Sri Lanka, the “series” used in Europe or North America have been adopted in whole but so far its effectiveness in isolating the triggers in local patients has not been assessed.

This study aimed to assess the success of different standard series used to identify triggers in contact dermatitis over the last 7 years from 2012 to 2018 in the National Hospital of Sri Lanka, which is the final dermatological referral point of undiagnosed patients in the whole country.

## Methods

This retrospective analysis of hospital records, focused on the results of patch testing carried out at the dermatology unit of the National Hospital of Sri Lanka (NHSL) over a period of 7 years from 2012 to 2018. The NHSL is the largest, premier public hospital in Sri Lanka and is the final referral point for all undiagnosed patients in dermatology and other clinical specialties. It is the only center that performed patch testing in the country during the period examined in this paper. The analysis presented here is a representative of the difficulty to diagnose cases of contact dermatitis in Sri Lanka.

The patch testing during this period has been done with three series: European Baseline Series, Shoe Series and the International Comprehensive Baseline Series (Additional file 1). Standard concentrations in the commercially available chemotechnique diagnostics patch testing kit were used for patch testing throughout the period without any change. Each patient has been tested with only one series, based on availability and the professional opinion of the consultant dermatologist overseeing patient care. The procedure is an epicutaneous diagnostic provocation test using standard haptens. Patch testing haptens are placed onto the patients’ skin and kept for 48 h and read after 48 h and 96 h. Irritant contact dermatitis was detected in several patients during patch testing as having positive results on first reading but being negative on second reading. They were considered as negative cases for patch testing, as our intention was to detect allergic contact dermatitis.

The patient records of patients who had undergone patch testing during this period (regardless of a positive result) were collected to an electronic database with available demographic (gender, age, occupation, ethnicity) and clinical data (duration of symptoms and patients perception of the irritant) included.

The database was analysed using SPSS statistical software (IBM, USA, Version 22). Descriptive statistics were summarized as measures of central tendency (mean or median) and measures of dispersion (standard deviation or inter-quartile range). Univariate analysis was carried out with chi-square test. Post-hoc analysis was carried out when there were multiple groups per variable in one comparison (degree of freedom ≥2). Statistically significant differences between proportions were calculated with z score test for proportions and the association between demographic factors and positivity to various allergens used in patch testing were assessed with binary logistic regression. Statistical significance was set at 0.05.

## Results

A total of 438 patients [mean age 45.6 years; age range, 14–82 years; 288 females (63%)] were tested. Of these, 307 (70.1%) had undergone EBS, 121 (27.6%) and 11 (2.3%) had undergone SS and ICBS respectively. All patients except one had been tested by only one series at the discretion of the consultant dermatologist. The mean number of allergens tested per patient was 28 (range 22–84). Among all patients, 239 (54.8%) showed at least one positive reaction and 212 (48%) had two or more positive reactions. When comparing the positivity rates by the series, ICBS had the highest positivity rate (90.9%, 10/11, at least one positive reaction per test) but it also had the lowest number of tested patients. Furthermore ICBS includes the highest number of tested substances. Between the other two series, the SS (70.2%, 85/121) had a significantly higher positivity rate than EBS (46.9%, 144/307, *p* < 0.05). Overall in 45.4% (199/438) of patients, the series used failed to generate a positive result. On a logistic regression analysis, there was no relationship between positivity by EBS or SS with patient age, ethnicity and gender. Regarding the year of testing, for EBS, 2018 had a significantly lower positive test rate compared to 2013, 2015, 2016 and 2017. For SS, both 2016 and 2017 had a significantly lower positivity rate compared to 2013. These findings persisted on the logistic regression analysis.

When all series were combined, nickel sulfate was the most frequently recognized contact allergen with a sensitization rate of 16% (70/438). This was followed by p-phenylenediamine (PPD) (12.3%, 54/438), 2-mercaptobenzothiazole (MBT) or mercapto mix (10.5%, 46/438), potassium dichromate (7.1%, 31/438) and thiuram mix (5.9%, 26/438). The top five sensitizers from SS and the EBS are listed in Table 1. We carried out a logistic regression analysis to see if age, year of testing, gender, ethnicity or the series used would have influenced a positive result for each of the top five allergens identified (Table 2). For nickel sulfate and thiuram mix no significant associations were identified. For PPD sensitivity, increasing age showed a positive association while year of testing showed a negative correlation (less likely to be positive in recent years). A similar association with year of testing was seen for MBT/mercapto mix as well. For potassium dichromate, males were more likely to be sensitive than females. For remaining allergens, regression analysis was not carried out, as the numbers of positive cases in each category were small.

**Table 1:** The five most common allergens / irritants in patch testing per series

Shoe Series (n-121)		European Baseline Series (n-307)	
Substance	Number (%)	Substance	Number (%)
MBT^a^	38 (44.7)	Nickel (II) sulfate	49 (16.0)
Nickel (II) sulfate	21 (24.7)	PPD^a^	42 (13.7)
Thiuram mix	16 (18.8)	Potassium dichromate	24 (7.8)
PTBP^a^	13 (15.3)	Cobalt (II) chloride	20 (6.5)
PPD^a^	12 (14.1)	Neomycin sulfate	15 (4.9)

^a^PPD- p-phenylenediamine (PPD), MBT −2-mercaptobenzothiazole, PTBP – 4-tert-butylphenolformaldehyde resin

**Table 2:** Factors associated with a positive result for the top five allergens/irritants identified by any series (adjusted analysis, showing significant results only)

Substance	Variable	*P* value	Exp(B) with 95% CI
Nickel sulfate	–	–	–
PPD^b^	Age^a^	0.015	1.03 (1.01–1.05)
	Year of testing^a^	0.011	0.79 (0.66–0.95)
MBT^b^ + Mercapto mix	Year of testing^a^	0.001	0.69 (0.56–0.87)
Potassium dichromate	Males (vs. females)	0.018	2.53 (1.17–5.49)
Thiuram mix	–	–	–

^a^per each increment of an year, ^b^PPD- p-phenylenediamine (PPD), MBT −2-mercaptobenzothiazole

## Discussion

This first retrospective analysis of patch testing results of Sri Lankan patients over a period of 7 years showed that an allergen was identified in approximately half of the patients tested. These patients were mostly tested by either the EBS or SS. The most common allergen in positive cases was nickel sulfate followed by PPD and MBT or mercapto mix.

The results of this study are comparable to those obtained by other authors who assessed the positivity rates of these standard series in other Asian countries. In a series of 240 consecutive patients in Saudi Arabia, 57% showed 1 or more positive results on patch testing (with EBS) with the most common allergen being nickel sulfate (51 = 37.5%) followed by potassium dichromate (48 = 35%) [3]. In Turkey 51.7% patients had at least one positive result (using an extended European standard series, supplemental series and their own substances) with nickel sulfate identified the most frequent sensitizer (19.1%), followed by potassium dichromate (11.8%) [4]. Other studies from China, India, and Israel report positivity rates between 43 and 65% of patients tested with EBS [5–7]. In a study in Thailand 81.2% patients (with 56.5% of clinical relevance) showed one or more positive reactions [8].

This raises an issue if these standard series need to be modified to fit the local populations in Asian countries. Though a perfectly optimized series is difficult to define given the wide range of common and unique allergens found within and between countries, inability to identify an allergen in almost half the people tested requires a coordinated effort to improve and validate a locally relevant patch testing series to increase yield.

With regard to the most common allergens, nickel sulfate frequently emerges as the top allergen in many Asian cohorts (in studies conducted in Saudi Arabia, China, Singapore, Israel and Turkey) [3–5, 7, 9]. This is closely followed by potassium dichromate with the exception of India where it takes the top spot [3–6, 10]. In Thailand gold sodium thiosulfate was the most frequent allergen [8] in one study and in another it was nickel sulfate [11]. Sensitivity to both nickel sulfate and potassium dichromate compounds was common in our patients with nickel sulfate taking the top spot but potassium dichromate was the fourth top allergen. Interestingly the latter was the only allergen of the top five sensitizers that had a significant gender bias with males more likely to be positive than females. It is a common ingredient in cement and males are more likely than females to be employed and exposed to cement in Sri Lanka. PPD was the second common allergen in our patients, which also showed a positive correlation with age but not with gender. It is a common constituent in hair dye with exposure more likely in older age groups than in younger patients.

## Conclusion

In this study, SS was tested on relatively fewer patients but it was more likely to yield a positive result than EBS. The ICBS had the highest positivity rate but it is more cumbersome to administer. Therefore looking to the future, it is prudent to use the SS as the preferred option in local patients and any modifications tailored to local populations can be done using this series. However it is noteworthy that positivity rates with both these series (as well as for some top sensitizers within them; PPD and MBT/mercapto mix) are on the decline. This is further evidence for the continuously changing nature of allergens in the environment and the need to modify existing patch testing standards accordingly. Retrospective and prospective analyses such as this study can form the foundation to develop locally useful, evidence based patch testing standards with a higher yield.

## Additional file

Additional file 1: The patch testing series. Standard concentrations in the commercially available chemotechnique diagnostics patch testing kit for European Baseline Series, Shoe Series and the International Comprehensive Baseline Series. The contents were taken from chemotechnique diagnostics patch test products and reference manual. (PDF 423 kb)

## Data Availability

The data set for this publication is not publically available and can be obtained from the corresponding author on request. The administrative permission to access the data was obtained from consultant dermatologist in National Hospital Of Sri lanka.

## Abbreviations

EBS: European Baseline Series
ICBS: International Comprehensive Baseline Series
MBT: 2-Mercaptobenzothiazole
PPD: P-Phenylenediamine
SS: Shoe Series
