# Supplementary material for: A seven-year retrospective analysis of patch test data in a cohort of patients with contact dermatitis in Sri Lanka
Source: BMC Dermatol. 2019 Jul 10;19:10. doi: 10.1186/s12895-019-0090-8 (PMC6617664; doi:10.1186/s12895-019-0090-8)
Supplement: Supplementary file 1 — The patch testing series. Standard concentrations in the commercially available chemotechnique diagnostics patch testing kit for European Baseline Series, Shoe Series and the International Comprehensive Baseline Series. The contents were taken from chemotechnique diagnostics patch test products and reference manual. (PDF 423 kb) [file 12895_2019_90_MOESM1_ESM.pdf]

CHEMOTECHNIQUE
DIAGNOSTICS

| Compound | Conc. %(w/w) | Veh. | Art. No. |
|---|---|---|---|
| **European Baseline Series** | | | **S-1000** |
| 1. Potassium dichromate | 0.5 | pet | P-014A |
| 2. p-PHENYLENEDIAMINE (PPD) | 1.0 | pet | P-006 |
| 3. Thiuram mix | 1.0 | pet | Mx-01 |
| -Dipentamethylenethiuram disulfide | 0.25 | | D-019 |
| -Tetraethylthiuram disulfide (TETD) | 0.25 | | T-002 |
| -Tetramethylthiuram disulfide (TMTD) | 0.25 | | T-005 |
| -Tetramethylthiuram monosulfide (TMTM) | 0.25 | | T-006 |
| 4. Neomycin sulfate | 20.0 | pet | N-001 |
| 5. Cobalt(II)chloride hexahydrate | 1.0 | pet | C-017A |
| 6. Benzocaine | 5.0 | pet | B-004 |
| 7. Nickel(II)sulfate hexahydrate | 5.0 | pet | N-002A |
| 8. Clioquinol | 5.0 | pet | C-015 |
| 9. COLOPHONIUM | 20.0 | pet | C-020 |
| 10. Paraben mix | 16.0 | pet | Mx-03C |
| -BUTYLPARABEN | 4.0 | | B-020 |
| -ETHYLPARABEN | 4.0 | | E-010 |
| -METHYLPARABEN | 4.0 | | M-012 |
| -PROPYLPARABEN | 4.0 | | P-020 |
| 11. N-Isopropyl-N-phenyl-4-phenylenediamine (IPPD) | 0.1 | pet | I-004 |
| 12. LANOLIN ALCOHOL | 30.0 | pet | W-001 |
| 13. Mercapto mix | 2.0 | pet | Mx-05A |
| -N-Cyclohexyl-2-benzothiazolesulfenamide | 0.5 | | C-023 |
| -2-Mercaptobenzothiazole (MBT) | 0.5 | | M-003 |
| -Dibenzothiazyl disulfide (MBTS) | 0.5 | | D-003 |
| -2-(4-Morpholinylmercapto)benzothiazol (MOR) | 0.5 | | M-016 |
| 14. Epoxy resin, Bisphenol A | 1.0 | pet | E-002 |
| 15. MYROXYLON PEREIRAE RESIN* | 25.0 | pet | B-001 |
| 16. 4-tert-Butylphenolformaldehyde resin (PTBP) | 1.0 | pet | B-024 |
| 17. 2-Mercaptobenzothiazole (MBT) | 2.0 | pet | M-003A |
| 18. FORMALDEHYDE | 2.0 | aq | F-002B |
| 19. Fragrance mix I* | 8.0 | pet | Mx-07 |
| -AMYL CINNAMAL | 1.0 | | A-014 |

* Emulsifier: SORBITAN SESQUIOLEATE 5%

| | Compound | Conc. Veh. %(w/w) | | Art. No. |
|---|---|---|---|---|
| | -CINNAMYL ALCOHOL | 1.0 | | C-013 |
| | -CINNAMAL | 1.0 | | C-014 |
| | -EUGENOL | 1.0 | | E-016 |
| | -GERANIOL | 1.0 | | G-001 |
| | -HYDROXYCITRONELLAL | 1.0 | | H-008 |
| | -ISOEUGENOL | 1.0 | | I-002 |
| | -Oakmoss absolute | 1.0 | | O-001 |
| 20. | Sesquiterpene lactone mix | 0.1 | pet | Mx-18 |
| | -Alantolactone | 0.033 | | A-003 |
| | -Costunolide | 0.033 | | C-039 |
| | -Dehydrocostus lactone | 0.033 | | D-056 |
| 21. | QUATERNIUM-15 | 1.0 | pet | C-007A |
| 22. | 2-Methoxy-6-n-pentyl-4-benzoquinone | 0.01 | pet | M-008 |
| 23. | METHYLISOTHIAZOLINONE + METHYLCHLOROISOTHIAZOLINONE | 0.01 | aq | C-009A |
| 24. | Budesonide | 0.01 | pet | B-033B |
| 25. | Tixocortol-21-pivalate | 0.1 | pet | T-031B |
| 26. | METHYLDIBROMO GLUTARONITRILE | 0.5 | pet | D-049E |
| 27. | Fragrance mix II | 14.0 | pet | Mx-25 |
| | -Hexyl cinnamic aldehyde | 5.0 | | H-025 |
| | -COUMARIN | 2.5 | | C-038 |
| | -FARNESOL | 2.5 | | F-004 |
| | -Lyral | 2.5 | | L-003 |
| | -CITRAL | 1.0 | | C-036 |
| | -CITRONELLOL | 0.5 | | C-037 |
| 28. | Lyral | 5.0 | pet | L-003 |
| 29. | METHYLISOTHIAZOLINONE | 0.2 | aq | M-035B |

Revised January 2014

It is strongly recommended to make an additional reading of the test on day 7.

References:

1. *M. Isaksson, F.M. Brandao, M.Bruze, A.Goossens. Recommendation to include budesonide and tixocortol privalate in the european standard series. Contact dermatitis 2000, 43, 41-42.*

2. *Maureen J. Jonker & Derk P. Bruynzeel. The outcome of an additional test reading on days 6 or 7. Contact dermatitis 2000, 42, 330-335.*

3. *Recommendation to include Fragrance mix II and hydroxyisohexyl 3-cyclohexene car-*

| **Compound** | **Conc. Veh. %(w/w)** | **Art. No.** |
|---|---|---|

*boxaldehyde (Lyral®) in Contact Dermatitis Volume 58, Issue 3, March 2008, Pages: 129–133, Magnus Bruze, Klaus Ejner Andersen and An Goossens, on behalf of the ESCD and EECDRG*

**Various national baseline series developed by the national contact dermatitis research groups are also available on request.**

## International Comprehensive Baseline Series ICB-1000

| | | | | |
|---|---|---|---|---|
| 1. | Benzocaine* | 5.0 | pet | B-004 |
| 2. | 2-Mercaptobenzothiazole (MBT) | 1.0 | pet | M-003B |
| 3. | COLOPHONIUM* | 20.0 | pet | C-020 |
| 4. | p-PHENYLENEDIAMINE (PPD)* | 1.0 | pet | P-006 |
| 5. | IMIDAZOLIDINYL UREA | 2.0 | pet | I-001A |
| 6. | CINNAMAL | 1.0 | pet | C-014 |
| 7. | Amerchol L 101 | 50.0 | pet | A-004 |
| 8. | Carba mix | 3.0 | pet | Mx-06 |
| | -1,3-Diphenylguanidine | 1.0 | | D-022 |
| | -ZINC DIBUTYLDITHIOCARBAMATE (ZBC) | 1.0 | | Z-002 |
| | -Zinc diethyldithiocarbamate (ZDC) | 1.0 | | Z-003 |
| 9. | Neomycin sulfate* | 20.0 | pet | N-001 |
| 10. | Thiuram mix* | 1.0 | pet | Mx-01 |
| | -Dipentamethylenethiuram disulfide | 0.25 | | D-019 |
| | -Tetraethylthiuram disulfide (TETD) | 0.25 | | T-002 |
| | -Tetramethylthiuram disulfide (TMTD) | 0.25 | | T-005 |
| | -Tetramethylthiuram monosulfide (TMTM) | 0.25 | | T-006 |
| 11. | Clobetasol-17-propionate | 1.0 | pet | C-028 |
| 12. | Ethylenediamine dihydrochloride | 1.0 | pet | E-005 |
| 13. | Epoxy resin, Bisphenol A* | 1.0 | pet | E-002 |
| 14. | QUATERNIUM-15* | 2.0 | pet | C-007B |
| 15. | 4-tert-Butylphenolformaldehyde resin (PTBP)* | 1.0 | pet | B-024 |
| 16. | Mercapto mix | 1.0 | pet | Mx-05B |
| | -N-Cyclohexyl-2-benzothiazolesulfenamide | 0.25 | | C-023 |
| | -2-Mercaptobenzothiazole (MBT) | 0.25 | | M-003 |

* Also present in European Baseline Series

| | Compound | Conc. %(w/w) | Veh. | Art. No. |
|---|---|---|---|---|
| | -Dibenzothiazyl disulfide (MBTS) | 0.25 | | D-003 |
| | -2-(4-Morpholinylmercapto)benzothiazol (MOR) | 0.25 | | M-016 |
| 17. | N-Isopropyl-N-phenyl-4-phenylenediamine (IPPD)* | 0.1 | pet | I-004 |
| 18. | Potassium dichromate | 0.25 | pet | P-014B |
| 19. | MYROXYLON PEREIRAE RESIN*,** | 25.0 | pet | B-001 |
| 20. | Nickel(II)sulfate hexahydrate | 2.5 | pet | N-002B |
| 21. | DIAZOLIDINYL UREA | 1.0 | pet | D-044C |
| 22. | TOCOPHEROL | 100 | | T-036 |
| 23. | Bacitracin | 20.0 | pet | B-032B |
| 24. | Mixed dialkyl thiourea | 1.0 | pet | Mx-24 |
| | -N,N´-Dibutylthiourea | 0.5 | | D-038 |
| | -N,N´-Diethylthiourea | 0.5 | | D-039 |
| 25. | DISPERSE ORANGE 3 | 1.0 | pet | D-032 |
| 26. | Paraben mix | 12.0 | pet | Mx-03A |
| | -BUTYLPARABEN | 3.0 | | B-020 |
| | -ETHYLPARABEN | 3.0 | | E-010 |
| | -METHYLPARABEN | 3.0 | | M-012 |
| | -PROPYLPARABEN | 3.0 | | P-020 |
| 27. | METHYLDIBROMO GLUTARONITRILE* | 0.5 | pet | D-049E |
| 28. | Fragrance mix I*,** | 8.0 | pet | Mx-07 |
| | -AMYL CINNAMAL | 1.0 | | A-014 |
| | -CINNAMYL ALCOHOL | 1.0 | | C-013 |
| | -CINNAMAL | 1.0 | | C-014 |
| | -EUGENOL | 1.0 | | E-016 |
| | -GERANIOL | 1.0 | | G-001 |
| | -HYDROXYCITRONELLAL | 1.0 | | H-008 |
| | -ISOEUGENOL | 1.0 | | I-002 |
| | -Oakmoss absolute | 1.0 | | O-001 |
| 29. | GLUTARAL** | 0.5 | pet | G-003B |
| 30. | 2-BROMO-2-NITROPROPANE-1,3-DIOL | 0.5 | pet | B-015B |
| 31. | Sesquiterpene lactone mix* | 0.1 | pet | Mx-18 |
| | -Alantolactone | 0.033 | | A-003 |
| | -Costunolide | 0.033 | | C-039 |
| | -Dehydrocostus lactone | 0.033 | | D-056 |

* Also present in European Baseline Series

** Emulsifier: SORBITAN SESQUIOLEATE 5%

| | Compound | Conc. %(w/w) | Veh. | Art. No. |
|---|---|---|---|---|
| 32. | THIMEROSAL | 0.1 | pet | T-007 |
| 33. | PROPOLIS | 10.0 | pet | P-022 |
| 34. | BENZOPHENONE-3 | 10.0 | pet | H-014C |
| 35. | CHLOROXYLENOL (PCMX) | 1.0 | pet | C-010B |
| 36. | Ethyleneurea, melamine formaldehyde mix** | 5.0 | pet | Mx-16 |
| | -Dimethylol dihydroxy ethylene urea | 4.0 | | D-012 |
| | -Melamine formaldehyde | 1.0 | | M-001 |
| 37. | 2-tert-Butyl-4-methoxyphenol (BHA) | 2.0 | pet | B-022 |
| 38. | Gold(I)sodium thiosulfate dihydrate | 0.5 | pet | G-005A |
| 39. | Ethyl acrylate | 0.1 | pet | E-004 |
| 40. | GLYCERYL THIOGLYCOLATE | 1.0 | pet | G-004 |
| 41. | Toluenesulfonamide formaldehyde resin | 10.0 | pet | T-010 |
| 42. | Methyl methacrylate | 2.0 | pet | M-013 |
| 43. | Cobalt(II)chloride hexahydrate* | 1.0 | pet | C-017A |
| 44. | Tixocortol-21-pivalate* | 0.1 | pet | T-031B |
| 45. | Budesonide* | 0.01 | pet | B-033B |
| 46. | COCAMIDE DEA | 0.5 | pet | C-019 |
| 47. | TRIETHANOLAMINE | 2.0 | pet | T-016 |
| 48. | Hydrocortisone-17-butyrate | 1.0 | pet | H-021B |
| 49. | Tea Tree Oil oxidized | 5.0 | pet | T-035B |
| 50. | Fragrance mix II* | 14.0 | pet | Mx-25 |
| | -Hexyl cinnamic aldehyde | 5.0 | | H-025 |
| | -COUMARIN | 2.5 | | C-038 |
| | -FARNESOL | 2.5 | | F-004 |
| | -Lyral | 2.5 | | L-003 |
| | -CITRAL | 1.0 | | C-036 |
| | -CITRONELLOL | 0.5 | | C-037 |
| 51. | Disperse Yellow 3 | 1.0 | pet | D-036 |
| 52. | BENZYL SALICYLATE | 10.0 | pet | B-010B |
| 53. | DECYL GLUCOSIDE*** | 5.0 | pet | D-065 |
| 54. | METHYLISOTHIAZOLINONE* | 0.2 | aq | M-035B |
| 55. | 2-Hydroxyethyl methacrylate | 2.0 | pet | H-010 |
| 56. | DMDM HYDANTOIN*** | 1.0 | pet | D-047B |

* Also present in European Baseline Series

** Emulsifier: SORBITAN SESQUIOLEATE 5%

*** Emulsifier: SORBITAN SESQUIOLEATE 1%

| | Compound | Conc. %(w/w) | Veh. | Art. No. |
|---|---|---|---|---|
| 57. | CANANGA ODORATA OIL | 2.0 | pet | Y-001 |
| 58. | BENZYL ALCOHOL | 10.0 | sof | B-008B |
| 59. | ISOPROPYL MYRISTATE | 20.0 | pet | I-003 |
| 60. | TRICLOSAN | 2.0 | pet | T-014 |
| 61. | Desoximetasone | 1.0 | pet | D-057 |
| 62. | POLYSORBATE 80 | 5.0 | pet | P-013 |
| 63. | IODOPROPYNYL BUTYLCARBAMATE | 0.2 | pet | I-008C |
| 64. | 2-n-Octyl-4-isothiazolin-3-one | 0.1 | pet | O-004 |
| 65. | Disperse Blue mix 106/124 | 1.0 | pet | Mx-26 |
| | -Disperse Blue 106 | 0.5 | | D-040 |
| | -Disperse Blue 124 | 0.5 | | D-041 |
| 66. | Compositae mix II | 5.0 | pet | Mx-29A |
| | -ANTHEMIS NOBILIS EXTRACT | 1.2 | | C-029 |
| | -CHAMOMILLA RECUTITA EXTRACT | 1.2 | | C-051 |
| | -ACHILLEA MILLEFOLIUM EXTRACT | 1.0 | | A-025 |
| | -TANACETUM VULGARE EXTRACT | 1.0 | | T-033 |
| | -ARNICA MONTANA EXTRACT | 0.5 | | A-024 |
| | -Parthenolide | 0.1 | | P-029 |
| 67. | Lidocaine | 15.0 | pet | L-002B |
| 68. | Fusidic acid sodium salt | 2.0 | pet | F-003 |
| 69. | Dibucaine hydrochloride | 2.5 | pet | D-005B |
| 70. | Benzoylperoxide | 1.0 | pet | B-007 |
| 71. | ISOAMYL p-METHOXYCINNAMATE | 10.0 | pet | I-009 |
| 72. | Lyral* | 5.0 | pet | L-003 |
| 73. | ETHYLHEXYL SALICYLATE | 5.0 | pet | O-007A |
| 74. | BENZALKONIUM CHLORIDE | 0.1 | aq | B-027 |
| 75. | Amidoamine | 0.1 | aq | A-029 |
| 76. | COCAMIDOPROPYL BETAINE | 1.0 | aq | C-018 |
| 77. | FORMALDEHYDE* | 2.0 | aq | F-002B |
| 78. | METHYLISOTHIAZOLINONE + METHYLCHLOROISOTHIAZOLINONE* | 0.01 | aq | C-009A |
| 79. | PROPYLENE GLYCOL | 30.0 | aq | P-019B |
| 80. | Dimethylol dihydroxy ethylene urea | 4.5 | aq | D-012 |
| 81. | Hydroperoxides of Linalool | 1.0 | pet | H-031 |
| 82. | Hydroperoxides of Limonene | 0.3 | pet | H-032 |

Revised January 2014

* Also present in European Baseline Series

| | | | | |
|---|---|---|---|---|
| 1. | N-Isopropyl-N-phenyl-4-phenylenediamine (IPPD)* | 0.1 | pet | I-004 |
| 2. | GLUTARAL** | 0.2 | pet | G-003A |
| 3. | DISPERSE ORANGE 3 | 1.0 | pet | D-032 |
| 4. | Acid yellow 36 | 1.0 | pet | A-019 |
| 5. | Hydroquinone monobenzylether | 1.0 | pet | H-019 |
| 6. | Thiuram mix* | 1.0 | pet | Mx-01 |
| | -Dipentamethylene | 0.25 | | D-019 |
| | -Tetraethylthiuram | 0.25 | | T-002 |
| | -Tetramethylthiurar | 0.25 | | T-005 |
| | -Tetramethylthiuram monosulfide (TMTM) | 0.25 | | T-006 |
| 7. | Potassium dichromate* | 0.5 | pet | P-014A |
| 8. | 4-tert-Butylphenolformaldehyde resin (PTBP)* | 1.0 | pet | B-024 |
| 9. | p-PHENYLENEDIAMINE (PPD)* | 1.0 | pet | P-006 |
| 10. | Nickel(II)sulfate hexahydrate* | 5.0 | pet | N-002A |
| 11. | COLOPHONIUM* | 20.0 | pet | C-020 |
| 12. | FORMALDEHYDE* | 2.0 | aq | F-002B |
| 13. | N,N´-Diphenylthiourea (DPTU) | 1.0 | pet | D-025 |
| 14. | 2-Mercaptobenzothiazole (MBT)* | 2.0 | pet | M-003A |
| 15. | N,N´-Diethylthiourea | 1.0 | pet | D-039 |
| 16. | 1,3-Diphenylguanidine | 1.0 | pet | D-022 |
| 17. | N,N´-Dibutylthiourea | 1.0 | pet | D-038 |
| 18. | Epoxy resin, Bisphenol A* | 1.0 | pet | E-002 |
| 19. | Dodecyl mercaptan | 0.1 | pet | D-043 |
| 20. | METHYLISOTHIAZOLINONE + METHYLCHLORO-ISOTHIAZOLINONE | 0.02 | aq | C-009B |
| 21. | 4-Aminoazobenzene | 0.25 | pet | A-005 |
| 22. | 2-n-Octyl-4-isothiazolin-3-one | 0.1 | pet | O-004 |
| 23. | 4,4`-Dithiodimorpholine | 1.0 | pet | D-054 |

Revised January 2014

* Also present in European Baseline Series

** Emulsifier: SORBITAN SESQUIOLEATE 5%



[illegible]
